# Cyclosporine A reduces microvascular obstruction and preserves left ventricular function deterioration following myocardial ischemia and reperfusion

**DOI:** 10.1007/s00395-015-0475-8

**Published:** 2015-02-27

**Authors:** Jaroslaw Zalewski, Piet Claus, Jan Bogaert, Nina Vanden Driessche, Ronald B. Driesen, Diogo T. Galan, Karin R. Sipido, Piotr Buszman, Krzysztof Milewski, Frans Van de Werf

**Affiliations:** 1 Cardiology, Department of Cardiovascular Sciences, KU Leuven, Herestraat 49, 3000 Leuven, Belgium; 2 Department of Coronary Heart Disease, Jagiellonian University, Pradnicka Street 80, 31-202 Krakow, Poland; 3 Imaging and Cardiovascular Dynamics, Department of Cardiovascular Sciences, KU Leuven, Leuven, Belgium; 4 Radiology, UZ Leuven, Translational MRI, Department of Imaging and Pathology, KU Leuven, Leuven, Belgium; 5 Experimental Cardiology, Department of Cardiovascular Sciences, KU Leuven, Leuven, Belgium; 6 Center for Cardiovascular Research and Development, American Heart of Poland, Ustron, Poland

**Keywords:** Myocardial infarction, Cyclosporine A, Postconditioning, Cardiovascular magnetic resonance imaging, Microvascular obstruction

## Abstract

Postconditioning and cyclosporine A prevent mitochondrial permeability transition pore opening providing cardioprotection during ischemia/reperfusion. Whether microvascular obstruction is affected by these interventions is largely unknown. Pigs subjected to coronary occlusion for 1 h followed by 3 h of reperfusion were assigned to control (*n* = 8), postconditioning (*n* = 9) or cyclosporine A intravenous infusion 10–15 min before the end of ischemia (*n* = 8). Postconditioning was induced by 8 cycles of repeated 30-s balloon inflation and deflation. After 3 h of reperfusion magnetic resonance imaging, triphenyltetrazolium chloride/Evans blue staining and histopathology were performed. Microvascular obstruction (MVO, percentage of gadolinium-hyperenhanced area) was measured early (3 min) and late (12 min) after contrast injection. Infarct size with double staining was smaller in cyclosporine (46.2 ± 3.1 %, *P* = 0.016) and postconditioning pigs (47.6 ± 3.9 %, *P* = 0.008) versus controls (53.8 ± 4.1 %). Late MVO was significantly reduced by cyclosporine (13.9 ± 9.6 %, *P* = 0.047) but not postconditioning (23.6 ± 11.7 %, *P* = 0.66) when compared with controls (32.0 ± 16.9 %). Myocardial blood flow in the late MVO was improved with cyclosporine versus controls (0.30 ± 0.06 vs 0.21 ± 0.03 ml/g/min, *P* = 0.002) and was inversely correlated with late-MVO extent (*R* ^*2*^ = 0.93, *P* < 0.0001). Deterioration of left ventricular ejection fraction (LVEF) between baseline and 3 h of reperfusion was smaller with cyclosporine (−7.9 ± 2.4 %, *P* = 0.008) but not postconditioning (−12.0 ± 5.5 %, *P* = 0.22) when compared with controls (−16.4 ± 5.5 %). In the three groups, infarct size (*β* = −0.69, *P* < 0.001) and late MVO (*β* = −0.33, *P* = 0.02) were independent predictors of LVEF deterioration following ischemia/reperfusion (*R* ^*2*^ = 0.73, *P* < 0.001). Despite both cyclosporine A and postconditioning reduce infarct size, only cyclosporine A infusion had a beneficial effect on microvascular damage and was associated with better preserved LV function when compared with controls.

**Electronic supplementary material:**

The online version of this article (doi:10.1007/s00395-015-0475-8) contains supplementary material, which is available to authorized users.

## Introduction

Despite complete epicardial blood flow restoration during primary coronary angioplasty in acute myocardial infarction, ischemia/reperfusion is associated with cardiomyocyte necrosis and with damage of coronary microvasculature and interstitial edema [6, 22, 23] which lead to lack of adequate tissue perfusion referred to as no-reflow phenomenon [32]. Even a small amount of microvascular damage can be detected by cardiovascular magnetic resonance (CMR) as microvascular obstruction (MVO) which is defined as a dark zone with impaired contrast wash-in within the high-signal-intensity infarcted region on early (3–5 min after contrast injection) or late (>10 min post-contrast) gadolinium-enhanced images [5]. The histopathological characteristics of microvascular obstruction have been extensively studied [46] and its occurrence in patients is associated with scar formation, left ventricular remodeling and worse clinical outcome [42, 53]. Thus, the reduction of microvascular damage seems to be an important target for adjunctive treatment besides reperfusion [24, 31].

The opening of mitochondrial permeability transition pore (mPTP) is considered a key event in cell death after ischemia and reperfusion [9, 31]. Mitochondrial permeability transition pore that could not open in the acidic milieu during ischemia, quickly opens as pH rises back to the neutral level during reperfusion [2] and leads to collapse of the mitochondrial transmembrane potential, cessation of adenosine triphosphate production and subsequent cell death [15]. Postconditioning composed of several brief cycles of ischemia alternating with reperfusion applied immediately after relief of a prolonged coronary occlusion, prevents mitochondrial permeability transition pore opening by maintaining acidosis at the onset of reperfusion [8], triggers complex molecular pathways in the myocardium and may reduce infarct size [25, 48, 49, 56]. However, a benefit of postconditioning on infarct size has not been shown in all animal models [11, 19, 27, 47]. Recently, it has been reported that postconditioning in patients with myocardial infarction reduces infarct size [51] and microvascular obstruction [38], however, in both studies the lower area at risk at baseline in postconditioning patients and the frequent use of aspiration thrombectomy were potential confounders. Moreover, in large randomized clinical trial postconditioning applied during primary angioplasty did not improve reperfusion and clinical outcome [18]. It has been proposed that cyclosporine can recapitulate the beneficial effects of postconditioning and a systematic review of experimental studies has shown that cyclosporine A infused during early reperfusion variably and inconsistently reduces infarct size [35].

Whether cyclosporine A or postconditioning could also have a beneficial effect on microvascular damage remains unknown. Clinical studies in this regard have limitations as the timing of CMR is not always optimal and correlation to histology is not possible. Therefore, we sought to investigate whether postconditioning or cyclosporine A infusion influence microvascular obstruction and its functional consequences in a pig model of coronary ischemia and reperfusion. If it turned out that cyclosporine A or postconditioning had a beneficial effect on microvascular obstruction, a series of additional experiments would be conducted to study their impact on myocardial edema, regional myocardial blood flow and the size of no-reflow area.

## Methods

### Animal preparation

All animals were treated according to National Health Guide for the Care and Use of Laboratory Animals and the study was approved by the Animal Care and Use Committee of the University of Leuven (reference number ECD P013/2009) and the Ethical Committee of the Institute of Pharmacology of the Polish Academy of Sciences (reference number 1111/2014).

Domestic pigs of both genders weighing 30–40 kg have been used. Pigs were pre-treated for 3 days with amiodarone (twice a day 200 mg) to reduce life-threatening arrhythmias. The day before the experiment they received 300 mg of aspirin and 300 mg of clopidogrel. Animals were pre-medicated with a combination of tiletamine and zolazepam (Zoletil^®^100, Virbac Switzerland SA) in a dose of 8 mg/kg intramuscularly (i.m.) and Xylazine 2.5 mg/kg i.m. (Vexylan^®^, CEVA Sante Animale, Belgium) before induction of anesthesia with propofol 3 mg/kg intravenously (i.v.) (Diprivan^®^ 1 %, AstraZeneca Canada Inc) and remifentanil 1 µg/kg i.v. (Ultiva^®^, GlaxoSmithKline, UK Ltd). After intubation, anesthesia was maintained with propofol 10 mg/kg/h i.v. And remifentanil 0.3 µg/kg/min i.v. During mechanical ventilation the gas mixture, tidal volume and ventilation rate were adjusted to maintain physiologic blood gases. Clinical examination including corneal reflex and response to pain was used to monitor the adequacy of anesthesia. An 8F catheter was introduced into the carotid artery to measure blood pressure and to access coronary arteries for angiography as well as for blood sampling. Hemodynamic recordings (Millar) were done at baseline and every 30 min during ischemia and reperfusion whereas ECG was continuously monitored. Data were processed using dedicated recording and analysis software (LabChart, ADInstruments, Oxford, UK).

### The experimental protocol

**Fig. 1:**
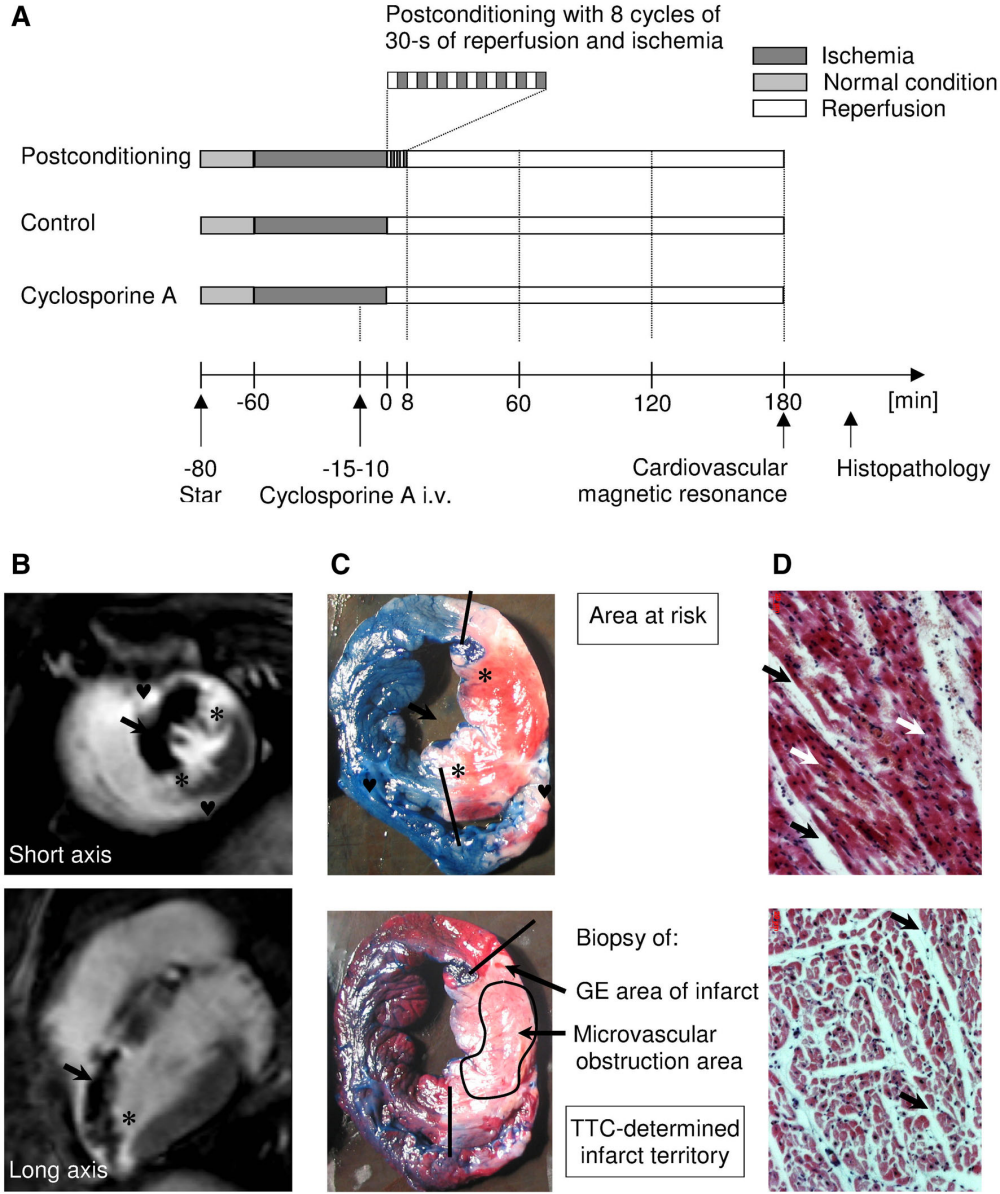
Methods. **a** Study protocol. **b** Gadolinium-enhanced (GE) images in short and horizontal long axis. The microvascular obstruction (indicated by *arrows*) is a dark zone in the high-intensity signal of infarct area. The left ventricle landmarks—place of insertion of papillary muscles (*asterisk*) and right ventricle (♥)—have been used to obtain biopsy specimens from the triphenyltetrazolium (TTC)-determined infarct territory. **c** Representative pictures of TTC/Evans blue staining with indicated place of biopsy corresponding with gadolinium-enhanced area of infarct and microvascular obstruction. **d** Microscopic pictures of infarct region with and without erythrocytes stasis. *Black arrows* indicate interstitial edema whereas *white arrows* show capillaries occupied by erythrocytes

A first series of animals were randomized to the control group (*n* = 8), the postconditioning group (*n* = 9) or the cyclosporine A group (*n* = 8) and were subjected to 60 min of ischemia and 180 min of reperfusion (Fig. 1a). Myocardial ischemia was induced by balloon inflation in the left anterior descending coronary artery, immediately after the origin of the first diagonal branch. In the control group no additional intervention was performed; in the postconditioning group 8 cycles of repeated 30-s balloon inflation and deflation induced no later than 30 s after index ischemia [27, 47]; in the cyclosporine A group, cyclosporine A (Sandimmune^®^, Novartis Pharmaceuticals Co, USA) in a dose of 10 mg per kilogram body weight was administered as a peripheral i.v. injection between 15 and 10 min prior to the end of myocardial ischemia to prevent mitochondrial permeability transition pore opening during reperfusion, as previously described [1, 35]. Both left anterior descending artery occlusion and its reopening were confirmed by contrast injection. After 3 h of reperfusion, CMR imaging was performed and then potassium chloride was given intravenously to induce cardiac arrest.

As microvascular obstruction was effectively reduced only with cyclosporine A infusion, using the same protocol a series of additional experiments was conducted in the cyclosporine A (*n* = 8) and control group (*n* = 8) (Online Resource 1). In these animals we aimed at elucidating the mechanisms of MVO reduction with cyclosporine A, by additionally comparing the size of myocardial edema, regional myocardial blood flow (MBF) as measured by CMR and the size of no-reflow territory as measured by the defect of thioflavin S staining.

### The assessment of arrhythmia and epicardial blood flow

The total time of sustained (>30 s) ventricular tachycardia and occurrence of ventricular fibrillation during ischemia and reperfusion were recorded. All coronary angiograms performed at baseline and at 15 min of reperfusion were analyzed off-line. Epicardial blood flow in left anterior descending artery as an infarct-related vessel and left circumflex artery as a non-infarct-related vessel was evaluated by means of the TIMI (Thrombolysis in Myocardial Infarction) and TIMI frame count scales.

### Cardiovascular magnetic resonance acquisition

**Fig. 2:**
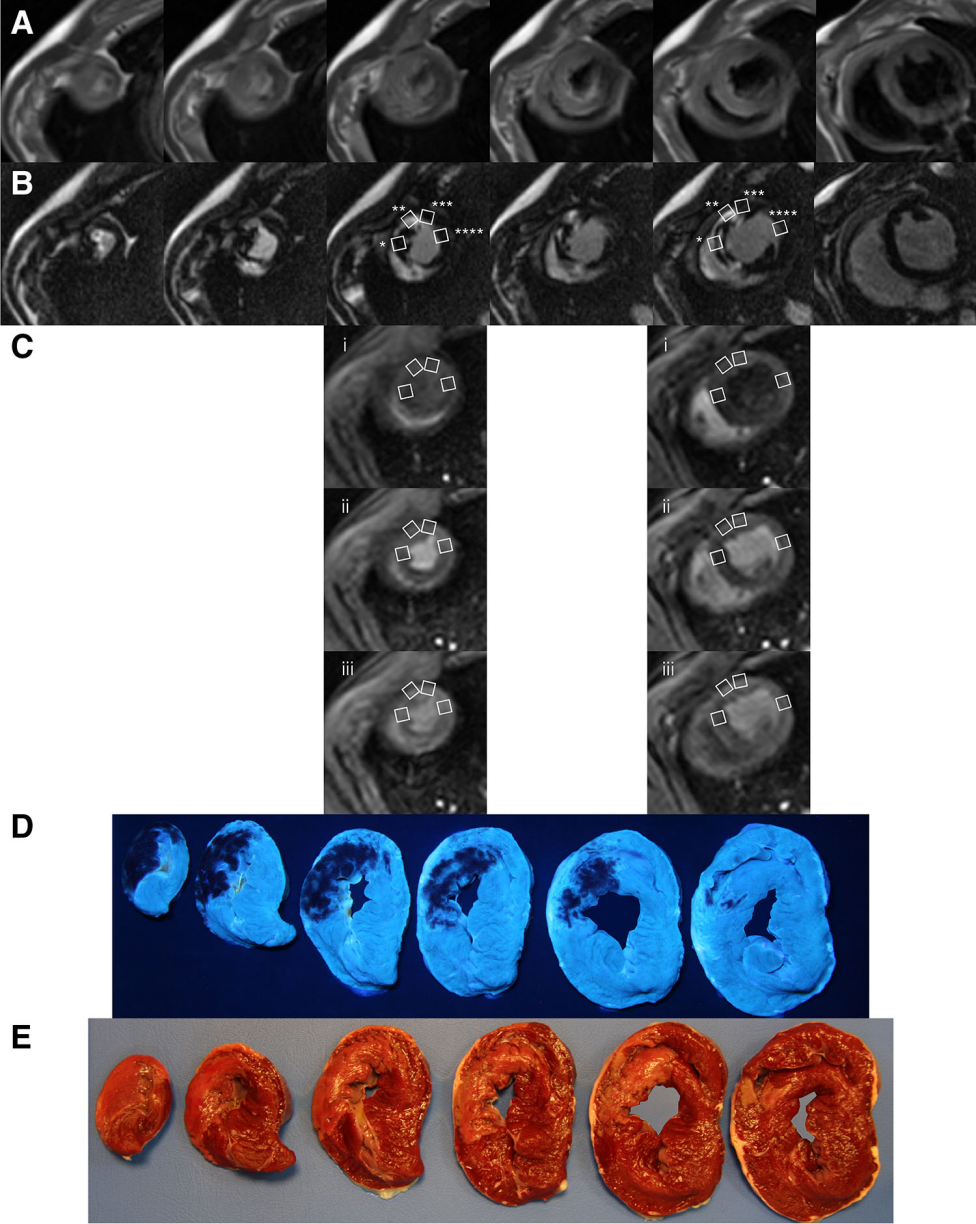
Cardiovascular magnetic resonance imaging and histopathology. Representative pictures of corresponding: **a** T2-weighted myocardial edema, **b** hyperenhanced area of infarct with dark zone of microvascular obstruction (MVO), **c** two perfusion slices of first pass with contrast medium in the right ventricle (i), in both ventricles and myocardium (ii) and in the left ventricle (iii), **d** no-reflow region as a lack of thioflavin S staining, **e** the infarct region determined by triphenyltetrazolium staining. *MVO region, **gadolinium-enhanced infarct area without MVO, ***border zone, ****remote myocardium

Cardiovascular magnetic resonance has been performed on a 3.0-T scanner (Magnetom Trio, a Tim System, Siemens, Erlangen, Germany) at baseline (on average 2 days before experiment) and after 3 h of reperfusion. Images were acquired with a phased array body coil wrapped over the heart, with electrocardiogram gating and during suspended respiration. After determination of cardiac axes with localizers, global and regional left ventricular function has been assessed with cine imaging in a vertical long axis and horizontal long axis plane as well as a stack of short-axis planes using a spoiled gradient echo sequence (TURBO-FLASH) with the following imaging parameters: repetition time 35.35 ms, echo time 2.47 ms, flip angle 12, field of view 330 × 268 mm, voxel size 1.7 × 1.7 × 6.0 mm, 40 cardiac phases, bandwidth 450 Hz/Px, thickness of slice 6 mm and gap 0 mm. Following cine sequences, gadolinium-enhanced imaging 3 (early) and 12 min (late) after contrast injections of 0.2 mmol/kg gadoterate meglumine (Dotarem, Guerbet, Roissy, France) was performed with a segmented three-dimensional inversion recovery with a gradient echo readout. Typical sequence parameters were: repetition time 2.19 ms, echo time 0.78 ms, flip angle 158, field of view 350 mm, voxel size 2.0 × 1.4 × 5.0 mm. The inversion time was modified iteratively to obtain maximal nulling of normal myocardium. For both cine and contrast-enhanced imaging, contiguous short-axis slices covered the entire left ventricle along its long axis from base to apex. In contrast with the dark-gray signal of the normal myocardium, the microvascular obstruction was identified as a dark zone, usually located in the subendocardium within the hyperenhanced, infarcted myocardium, on both early (3 min post-contrast) and late (12 min post-contrast) gadolinium-enhanced images (Figs. 1b, 2b, 4a, b) [5].

In additional experiments, apart from above-described cine and gadolinium-enhanced imaging (Figs. 1b, 2b), T2-weighted myocardial edema imaging (Fig. 2a) and first-pass perfusion technique (Fig. 2c) have been performed with 1.5-T scanner (Signa EXCITE, GE Healthcare, Milwaukee, WI, USA) after 3 h of reperfusion. The myocardial edema imaging was performed with the use of breath-hold, black blood, T2-weighted, double-inversion recovery fast spin echo sequence. In short-axis orientation, the left ventricle was entirely encompassed by 8-mm contiguous slices (Fig. 2a). Typical imaging parameters were: repetition time: 2 R–R intervals, echo time 62.88 ms, echo train length 32, field of view 380 × 380 mm, matrix 224 × 224 mm, thickness of slice 8 mm and gap 0 mm.

Subsequently, dual-bolus first-pass perfusion imaging was performed as previously shown [7, 26, 54]. Briefly, two gadobutrolum (Gadovist 1 mmol/ml, Bayer Pharma AG, Germany) doses of 0.0025 and 0.05 mmol/kg of equal volume of 10 ml were injected intravenously at a rate of 3.5 ml/s by CMR-compatible contrast injector and flushed by 20 ml of saline at the same rate. Perfusion slices were acquired at the two levels of insertion of papillary muscles to left ventricle and at the top of papillary muscle with s breath-hold fast gradient echo echo train sequence (Fig. 2c). Typical sequence parameters were: repetition time 6.56 ms, echo time 1.38 ms, echo train length 4, flip angle 20, field of view 400 × 400 mm and matrix 224 × 224 mm.

### Cardiac magnetic resonance analysis

All identifiers and image parameters were removed from the images before analysis. Images were analyzed blinded and in a random order on a post-processing workstation with dedicated software (Cardioviewer, KU Leuven, Belgium).

### Cardiac function, infarct size and microvascular obstruction

The end-diastolic and end-systolic phases were set up as the phase with the largest and smallest left ventricular volumes, respectively. Then the endocardial and epicardial borders were traced on the end-diastolic and end-systolic images to calculate left ventricular end-diastolic (LVEDV) and end-systolic (LVESV) volumes from which the left ventricular ejection fraction (LVEF), stroke volume and myocardial volume were calculated. Papillary muscles and trabeculations were included in the left ventricular cavity. The territory of infarct and microvascular obstruction was manually delineated on both the early and late gadolinium-enhanced images and volumes were calculated by multiplying the area of delineation with the thickness of the slice and gap. The mass of infarct, microvascular obstruction and left ventricle was obtained by multiplying respective volume by the specific density of myocardial tissue (1.05 g/ml). The hyperenhanced area of infarct was expressed as percentage of left ventricular mass and microvascular obstruction as percentage of infarct area. The area of microvascular obstruction was in the LGE area.

### Myocardial edema and regional myocardial blood flow

The territory of myocardial edema was determined in the same way as infarct area and was expressed as percentage of left ventricular mass. The regional myocardial blood flow analysis was described in detail previously [7, 54]. The time-signal intensity curves of LV cavity and the myocardial regions of interest generated after low and high contrast dose were used to calculate myocardial blood flow based on a Fermi function deconvolution method. Using the same localization of short-axis slices on corresponding gadolinium-enhanced images 12 min after contrast injection (Fig. 2b) and the two perfusion slices (Fig. 2c) regional myocardial blood flow in late-MVO region, gadolinium-hyperenhanced area without MVO, border zone and remote myocardium was determined.

### Histopathology analysis: area at risk, infarct size, no-reflow region and microscopy

As previously described [36], immediately after heart excision 2 % Evan’s blue was injected into the coronary ostia to outline the area at risk. The transversely sectioned left ventricular slices were incubated with 2,3,5-triphenyltetrazolium chloride (TTC, 1.4 %) at 37 °C to evaluate myocardial viability. Based on this double staining infarct territory (non-viable myocardium in the area at risk), border zone (viable myocardium in the area at risk) and remote myocardium were determined by planimetry using National Institutes of Health image software by one investigator blinded to the treatment group and cardiovascular magnetic resonance findings (Fig. 1c). Biopsies from these three areas were taken in the compared pigs. Additionally, using left ventricular landmarks (in long axis place of insertion of papillary muscles, in short-axis place of insertion of right ventricle) from triphenyltetrazolium-determined infarct territory, biopsy specimens corresponding with microvascular obstruction and hyperenhanced region were collected in 4 pigs from each study group (Fig. 1c). All biopsies were then fixed in 3 % paraformaldehyde during 24 h and routinely embedded in paraffin. 7.5-µm sections were stained with hematoxylin–eosin, dehydrated and mounted in Depex medium. The morphometric analysis was performed by two investigators blinded to the cardiovascular magnetic resonance imaging data on a Zeiss Axiovert 200 N microscope equipped with an Axiocam HrC camera. The contribution of cardiac myocytes, interstitial space and capillaries with erythrocytes quantitatively evaluated using Axiovision 4.6 morphometry software (Carl Zeiss, Oberkochen, Germany) was expressed as a percentage (Fig. 1d).

In a series of additional experiments, before heart excision 1 ml/kg of 4 % fluorescent thioflavin S dye was injected via pigtail catheter into the left ventricular cavity. After heart excision, the left ventricle was sectioned into 8-mm thick short-axis slices. To detect the area without thioflavin S staining reflecting the no-reflow region, each slice was viewed under ultraviolet light (Fig. 2d). Afterwards, slices were incubated with TTC (Fig. 2e). The no-reflow region expressed as the lack of thioflavin S staining within the TTC territory was determined by planimetry as above.

### Statistical analysis

The study was powered to have a 90 % chance to demonstrate a 25 % relative reduction of microvascular obstruction (absolute microvascular obstruction reduction from 20 to 15 % of infarct size) with a standard deviation derived from previous studies of ≤3 % using a *P* value of 0.05 [5, 36]. To demonstrate such a reduction, 8 pigs were required in each group.

Statistical analyses were performed with SPSS 20.0 software. For each data set, a box-whisker analysis was performed. Outliers, i.e. outside the 1.5 times interquartile range, were removed from the specific dataset and the derived measurements. Data are expressed as mean (± standard deviation) or median (interquartile range, IQR). Continuous variables were first checked for normal distribution by the Shapiro–Wilk statistics. Analysis of variance followed by a post hoc Bonferroni test was used to compare differences of single measurements in the three groups with normally distributed data whereas non-normally distributed data were analyzed by Kruskal–Wallis test and differences between groups were identified using a test for multiple comparisons of mean ranks. The association between two variables with a normal or non-normal distribution was assessed by the Pearson or Spearman test, respectively. Dependent normally or non-normally distributed variables were compared by *t* test for paired samples or Wilcoxon signed-rank test, respectively. Analysis of variance for repeated measures assuming sphericity was used to test serial hemodynamic data whereas analysis of covariance assuming homogeneity of slopes including the baseline value as a continuous covariate was used to compare microvascular obstruction, left ventricular function and epicardial blood flow changes from baseline in the three groups. The multiple linear regression analysis was used to determine predictors of LVEF deterioration following ischemia/reperfusion. For the additional experiments involving only controls and cyclosporine A group, student’s *t* test or Mann–Whitney *U* test were applied to compare differences between the two groups with normally or non-normally distributed data, respectively. A two-sided *P* < 0.05 was considered statistically significant.

## Results

### Clinical and hemodynamic implications of ischemia and reperfusion

Out of the 25 randomized pigs, one assigned to the postconditioning group died at 1 h and 20 min of reperfusion due to ventricular fibrillation resistant to defibrillation and was excluded from further analysis. Cyclosporine A, postconditioning and control pigs did not differ in body weight (36.1 ± 3.8, 36.1 ± 2.0 and 37.7 ± 3.6 kg, respectively, *P* = 0.53) and gender (male pigs 4/8, 6/8 and 4/8, respectively, *P* = 0.54). At baseline and at each time point during ischemia and reperfusion, there were no significant differences in the three groups with regard to heart rate, systolic, diastolic and mean blood pressure (Table 1). The total time of ventricular tachycardia during reperfusion was reduced by cyclosporine A infusion and postconditioning versus controls (2.1 ± 1.3 and 1.4 ± 0.6 vs 5.1 ± 0.7 min, respectively, *P* < 0.0001 for both treatment groups).

**Table 1:** Hemodynamic data

Min	Baseline	Ischemia		Reperfusion				
	0	30	60	30	60	90	120	150
Heart rate								
Con	99.1 ± 13.0	87.8 ± 18.0	81.8 ± 9.2	101.0 ± 13.9	88.5 ± 21.1	84.0 ± 19.8	79.4 ± 14.0	79.8 ± 18.8
PoC	102.6 ± 17.1	90.4 ± 18.8	90.4 ± 19.4	99.3 ± 19.0	106.0 ± 25.1	96.9 ± 20.6	85.0 ± 11.8	80.0 ± 7.3
CsA	93.2 ± 9.7	88.5 ± 11.1	86.6 ± 10.3	94.1 ± 14.1	91.6 ± 14.4	85.3 ± 13.5	83.8 ± 17.5	87.0 ± 16.0
*P* value*	0.40	0.95	0.47	0.67	0.22	0.32	0.72	0.55
Systolic blood pressure								
Con	100.3 ± 13.4	90.5 ± 12.4	90.5 ± 12.2	90.3 ± 9.8	98.6 ± 7.9	94.5 ± 6.1	91.6 ± 6.1	93.6 ± 5.2
PoC	102.1 ± 10.9	93.8 ± 11.5	97.4 ± 12.7	96.9 ± 7.6	98.3 ± 12.4	96.1 ± 8.1	95.6 ± 7.9	96.4 ± 11.2
CsA	103.6 ± 18.4	92.5 ± 13.2	98.4 ± 12.8	93.5 ± 16.6	96.6 ± 11.9	98.4 ± 14.7	101.3 ± 13.9	101.5 ± 14.9
*P* value*	0.90	0.87	0.41	0.55	0.93	0.76	0.17	0.38
Diastolic blood pressure								
Con	65.6 ± 11.4	62.0 ± 10.8	62.1 ± 11.2	61.0 ± 7.6	67.8 ± 8.7	62.8 ± 4.9	59.9 ± 4.1	61.1 ± 6.2
PoC	68.1 ± 8.4	63.8 ± 9.8	64.0 ± 10.7	62.3 ± 7.6	66.8 ± 10.0	68.1 ± 9.0	66.5 ± 12.1	66.6 ± 12.7
CsA	66.5 ± 12.1	61.6 ± 10.8	65.4 ± 13.5	66.0 ± 14.4	69.3 ± 13.3	66.9 ± 12.2	69.4 ± 9.8	68.6 ± 8.8
*P* value*	0.90	0.91	0.86	0.61	0.90	0.48	0.14	0.29
Mean blood pressure								
Con	80.3 ± 12.4	74.9 ± 11.5	76.3 ± 9.9	73.3 ± 6.8	78.6 ± 8.4	76.9 ± 5.8	76.5 ± 6.5	75.9 ± 5.7
PoC	82.5 ± 9.9	78.6 ± 11.0	79.1 ± 11.5	77.3 ± 6.9	82.4 ± 10.8	79.4 ± 8.1	80.6 ± 10.5	80.5 ± 12.3
CsA	81.6 ± 12.2	77.1 ± 11.5	79.9 ± 11.1	77.0 ± 12.5	79.9 ± 10.5	79.9 ± 11.7	83.5 ± 9.9	82.9 ± 8.8
*P* value*	0.93	0.80	0.78	0.63	0.75	0.77	0.33	0.33

Data are shown as mean ± standard deviation. ANOVA for repeated measures of differences in the three groups for heart rate: *F* = 1.38, *df* = 14, *P* = 0.17; systolic blood pressure: *F* = 0.62, *df* = 14, *P* = 0.85; diastolic blood pressure: *F* = 0.66, *df* = 14, *P* = 0.81 and mean blood pressure: *F* = 0.25, *df* = 14, *P* = 0.99

*Con* control group (*n* = 8), *PoC* postconditioning group (*n* = 8), *CsA* cyclosporine A group (*n* = 8)

* ANOVA for differences in the three groups in a single time point

### Angiographic characteristics

At baseline all animals had TIMI-3 flow. At 15 min of reperfusion TIMI-2 flow was found in 2 controls, 4 postconditioning and 1 cyclosporine A pig (*P* = 0.34). After 15 min of reperfusion TFC increased significantly both, in the infarct-related left anterior descending artery (*P* < 0.01) and in the left circumflex artery (*P* < 0.05) when compared with baseline but no differences were found between the 3 groups (Table 2).

**Table 2:** Angiographic data

	B	R15	Δ	ANCOVA
TIMI frame count in left anterior descending artery				
Con	25.5 ± 4.6	31.3 ± 3.8	5.8 ± 2.4	*F* = 0.98 *df* = 2 *P* = 0.39
PoC	23.8 ± 3.2	31.8 ± 6.7	8.0 ± 6.7	
CsA	24.0 ± 3.0	29.0 ± 3.1	5.0 ± 1.9	
* P* value*	0.59	0.48	0.36	
TIMI frame count in left circumflex artery				
Con	21.3 ± 2.0	24.5 ± 1.6	3.8 ± 2.4	*F* = 2.30 *df* = 2 *P* = 0.13
PoC	19.5 ± 2.1	26.8 ± 5.1	7.3 ± 6.6	
CsA	19.9 ± 2.8	22.9 ± 2.9	3.0 ± 2.1	
* P* value*	0.37	0.11	0.11	

Data are shown as mean ± standard deviation. ANCOVA with baseline value as a continuous covariate for differences between *B* and R15 in the three groups

*Con* control group (*n* = 8), *PoC* postconditioning group (*n* = 8), *CsA* cyclosporine A group (*n* = 8), *B* baseline, *R15* 15 min of reperfusion, *Δ* difference between R15 and B

* ANOVA for differences in the three groups in a single time point

### Myocardial infarct size

**Fig. 3:**
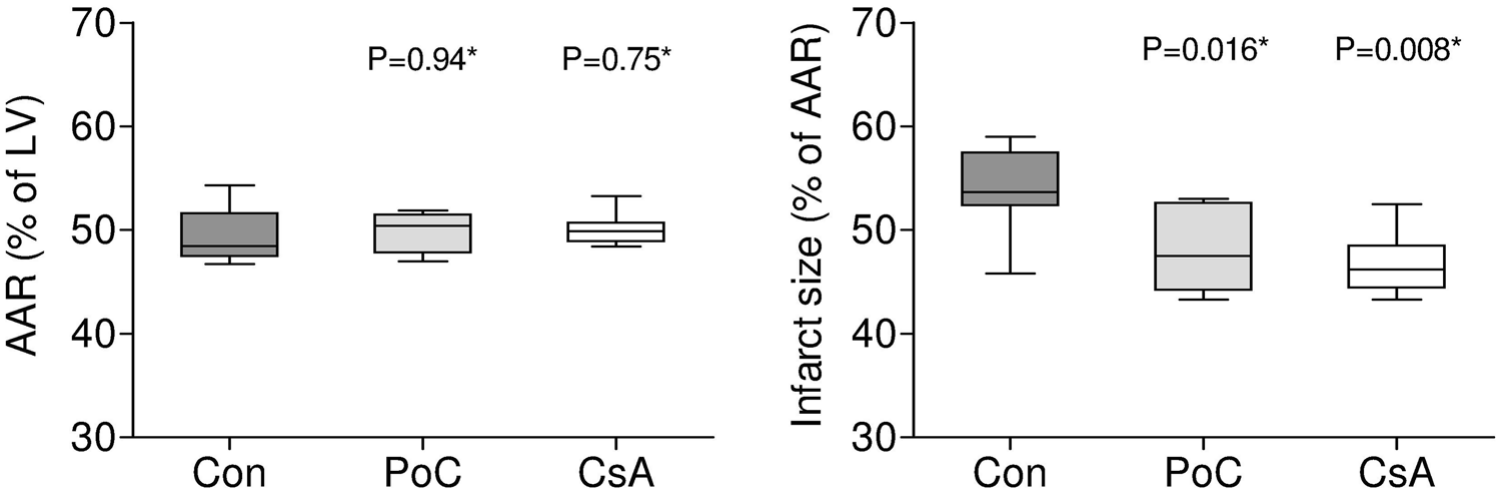
The area at risk and infarct size. Area at risk (AAR) by Evans blue and infarct size by triphenyltetrazolium staining, *box plot* shows median and interquartile range (IQR, Q3–Q1). Q1 and Q3 are the first and third quartiles. *Whiskers* are drawn at minimum and maximum. *LV* left ventricle, *Con* control group, *PoC* postconditioning group, *CsA* cyclosporine group, *versus controls

There were no differences between cyclosporine A, postconditioning and control pigs in the area at risk (50.2 ± 1.6, 49.8 ± 1.9 and 49.4 ± 2.6 %, respectively, *P* = 0.77 of ANOVA, Fig. 3).

Infarct size determined by double staining and expressed as percentage of area at risk was significantly lower in cyclosporine A (46.2 ± 3.1 %, *P* = 0.016) and in postconditioning pigs (47.6 ± 3.9 %, *P* = 0.008) as compared with control pigs (53.8 ± 4.1 %, Fig. 3). There was also a trend to a lower hyperenhanced area in cyclosporine A (24.3 ± 1.4 %, *P* = 0.20) and postconditioning pigs (23.0 ± 2.4 %, *P* = 0.022) versus controls (25.8 ± 1.2 %) at 12 min after contrast injection.

### Microvascular obstruction and its changes

**Fig. 4:**
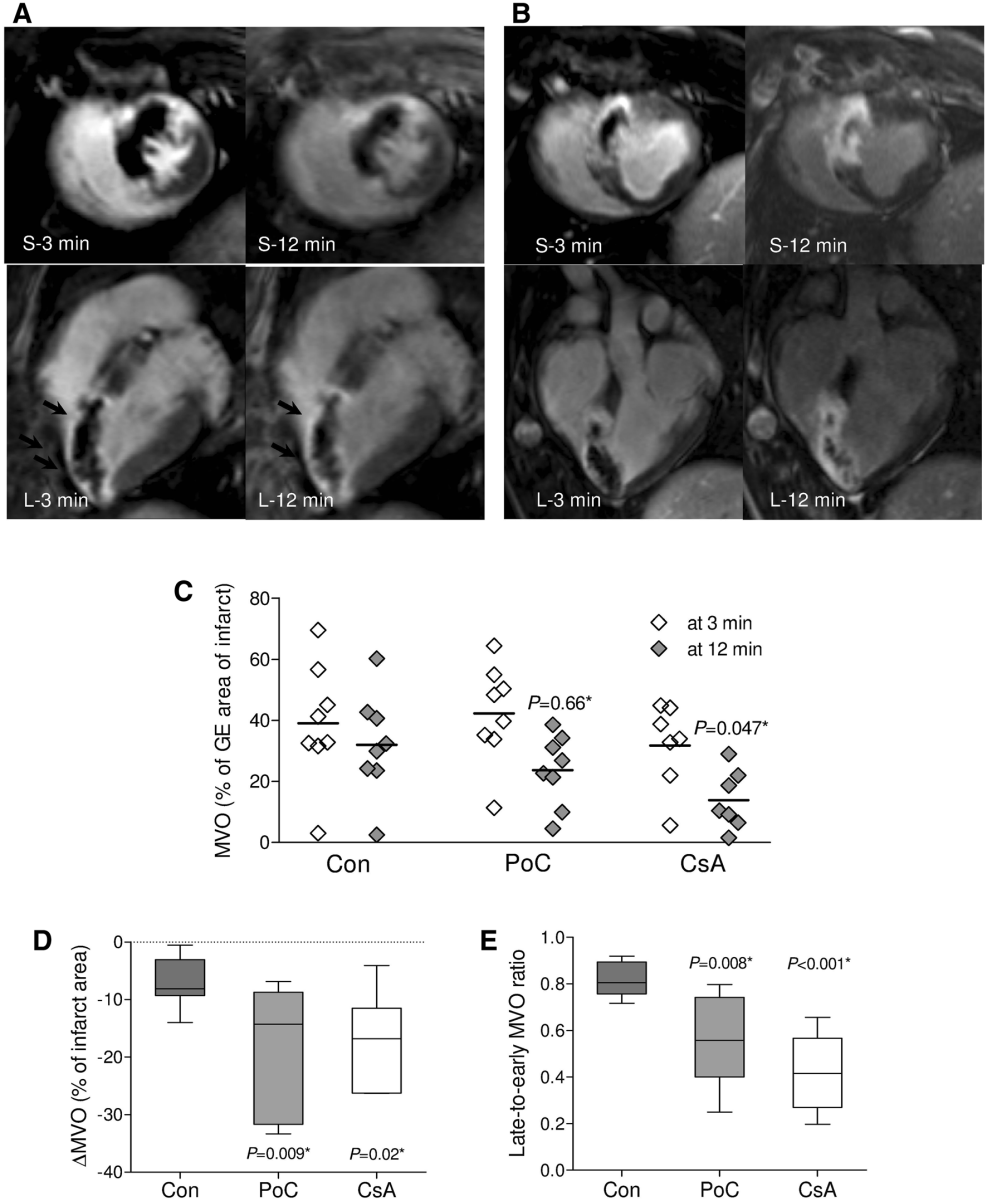
Microvascular obstruction and its dynamics. Gadolinium-enhanced (GE) images in short (S) and horizontal long (L) axis obtained at 3 (early) and 12 (late) min post-contrast. **a** Representative pictures of control pig with a small change of microvascular obstruction (MVO, indicated by *arrows*) extent by 25 % between 3 and 12 min (infarct size of 27 % of left ventricle). **b** Representative pictures of pig pre-treated with cyclosporine A with substantial decrease of MVO area by 60 % (infarct size of 25 % of left ventricle). **c** The early MVO and late MVO. *Scatter plot* shows absolute values and means. **d** The reduction of MVO area between 3 and 12 min post-contrast, **e** the late-to-early MVO ratio. *Box plot* shows median and interquartile range (IQR, Q3–Q1). Q1 and Q3 are the first and third quartiles. *Whiskers* are drawn at minimum and maximum, *Con* control group, *PoC* postconditioning group, *CsA* cyclosporine group. *versus controls

Microvascular obstruction at 3 (early) and at 12 min (late) after contrast injection was detected in all treated pigs (Fig. 4a and b, respectively). There were no significant differences in early MVO in the cyclosporine A, postconditioning and control groups (31.7 ± 13.9, 42.3 ± 16.2 and 39.1 ± 19.7 %, respectively, *P* = 0.48 of ANOVA) (Fig. 4c). Late MVO was significantly smaller in the cyclosporine A (13.9 ± 9.6 %, *P* = 0.047) but not in the postconditioning group (23.6 ± 11.7 %, *P* = 0.66) when compared with controls (32.0 ± 16.9 %, Fig. 4c). In the three groups, late MVO correlated with infarct size as measured by double staining (*R* = 0.44, *P* = 0.038) and with early MVO (*R* = 0.76, *P* < 0.001). Compared with control pigs, cyclosporine A infusion was associated with an absolute reduction of late MVO by 18.1 % (95 % CI 0.8–35.5 %) versus 8.4 % (95 % CI 8.4–25.2 %) for postconditioning.

Between 3 and 12 min after contrast injection MVO decreased in the three groups and these changes were independently associated with treatment assignment (*F* = 8.1, df = 2, *P* = 0.003) and with early MVO (*F* = 12.8, df = 1, *P* = 0.002). The decrease of MVO between 3 and 12 min after contrast was significantly higher in cyclosporine A (−17.9 ± 8.4 %, *P* = 0.02) and postconditioning (−18.6 ± 11.3, *P* = 0.009) versus control pigs (−7.1 ± 4.3, Fig. 4d). The ratio of late-to-early MVO was significantly lower in cyclosporine A (0.41 ± 0.17, *P* < 0.001) and postconditioning (0.55 ± 0.20, *P* = 0.008) versus control pigs (0.82 ± 0.07, Fig. 4e).

### Cardiac function and its determinants

**Fig. 5:**
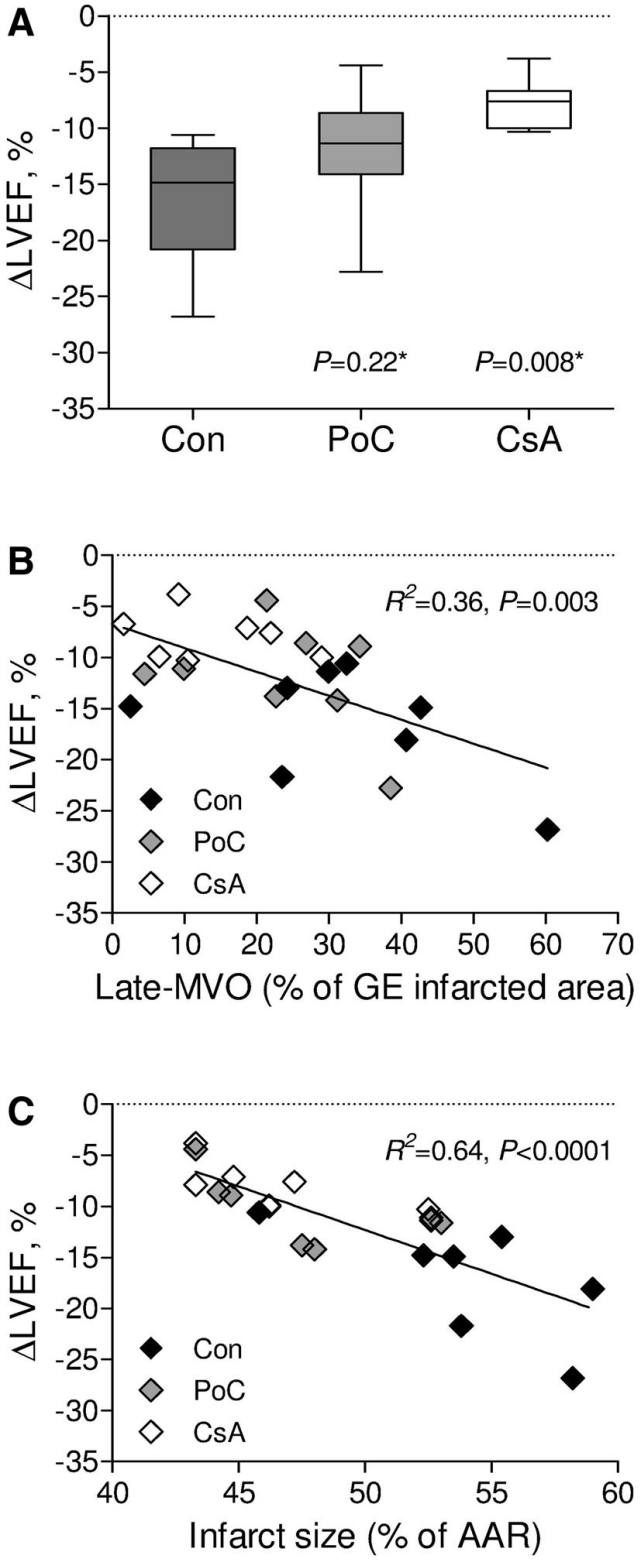
Changes of left ventricular ejection fraction and its determinants. **a** Changes of left ventricular ejection fraction between baseline and 3 h of reperfusion (LVEF). *Box plot* shows median and interquartile range (IQR, Q3–Q1). Q1 and Q3 are the first and third quartiles. *Whiskers* are drawn at minimum and maximum, *Con* control group, *PoC* postconditioning group, *CsA* cyclosporine A group, *versus controls. The relationship between: **b** LVEF and microvascular obstruction 12 min post-contrast (late MVO) or **c** LVEF and infarct size as percentage of area at risk (AAR) as measured by double staining

Left ventricular volumes, ejection fraction, left ventricular mass, stroke volume and cardiac output at different time points are given in Table 3. At 3 h of reperfusion an increase in LVESV and left ventricle mass and a decrease in LVEDV and cardiac output, without significant inter-group differences was found. Deterioration of LVEF between baseline and 3 h of reperfusion was limited by cyclosporine A infusion (−7.9 ± 2.4 %, *P* = 0.008) but not postconditioning (−11.9 ± 5.4 %, *P* = 0.22) when compared with controls (−16.4 ± 5.5 %, Fig. 5a) (Table 3). The LVEF deterioration after 3 h of reperfusion was inversely correlated with late MVO (Fig. 5b) and infarct size determined by double staining (Fig. 5c). Adjusted for left ventricle mass, both infarct size (*β* = −0.69, 95 % confidence interval for *β* [−0.34; −0.85], *P* < 0.001) as well as late MVO [*β* = −0.33, 95 % confidence interval for *β* (−0.12; −0.66), *P* = 0.02] were independent predictors of LVEF deterioration following ischemia/reperfusion. In total, the independent variables explained 73 % (*P* < 0.001) of variance associated with LVEF deterioration immediately after coronary occlusion and reperfusion, including 37 % of variance explained only by infarct size and 10 % by late MVO.

**Table 3:** Cardiac function

	B	R180	Δ	ANCOVA
LVEDV (ml)				
Con	78.0 ± 11.4	61.8 ± 6.4	−16.1 ± 9.8	*F* = 2.75 *df* = 2 *P* = 0.09
PoC	80.1 ± 11.9	71.7 ± 13.4	−8.4 ± 16.5	
CsA	78.5 ± 12.4	72.9 ± 11.2	−5.5 ± 5.4	
* P* value*	0.93	0.11	0.21	
LVESV (ml)				
Con	34.9 ± 5.1	37.7 ± 3.9	2.8 ± 4.5	*F* = 0.43 *df* = 2 *P* = 0.66
PoC	36.2 ± 5.6	40.7 ± 7.2	4.5 ± 6.5	
CsA	35.8 ± 5.2	39.0 ± 5.6	3.2 ± 3.4	
* P* value*	0.87	0.58	0.78	
LVEF (%)				
Con	55.3 ± 2.0	38.9 ± 5.6	−16.4 ± 5.5	*F* = 5.40 *df* = 2 *P* = 0.014
PoC	54.8 ± 1.5	42.9 ± 5.9	−11.9 ± 5.4	
CsA	54.2 ± 2.2	46.3 ± 3.5**	−7.9 ± 2.4**	
* P* value*	0.59	0.036	0.01	
SV (ml)				
Con	43.1 ± 6.7	24.1 ± 5.0	−19.0 ± 7.2	*F* = 4.66 *df* = 2 *P* = 0.023
PoC	43.9 ± 6.6	30.9 ± 7.9	−12.9 ± 10.9	
CsA	42.6 ± 7.6	33.9 ± 6.7**	−8.7 ± 2.8	
* P* value*	0.94	0.027	0.06	
CO (ml/s)				
Con	71.2 ± 10.9	42.9 ± 6.6	−28.3 ± 4.4	*F* = 1.31 *df* = 2 *P* = 0.29
PoC	72.1 ± 10.9	43.7 ± 6.6	−28.4 ± 4.3	
CsA	70.4 ± 12.5	42.6 ± 7.6	−27.7 ± 4.9	
* P* value*	0.96	0.96	0.96	
LV mass (g)				
Con	59.7 ± 8.8	75.8 ± 12.0	16.2 ± 7.3	*F* = 1.59 *df* = 2 *P* = 0.23
PoC	63.8 ± 5.9	74.6 ± 10.0	10.8 ± 6.6	
CsA	63.0 ± 7.4	73.3 ± 8.3	10.3 ± 6.7	
*P* value*	0.52	0.89	0.20	

Data are shown as mean ± standard deviation. ANCOVA with baseline value as a continuous covariate for differences between *B* and R180 in the three groups

*LV* left ventricle, *LVEDV* LV end-diastolic volume, *LVESV* LV end-systolic volume, *LVEF* LV ejection fraction, *CO* cardiac output, *SV* stroke volume, *Con* control group (*n* = 8), *PoC* postconditioning group (*n* = 8), *CsA* cyclosporine A group (*n* = 8), *B* baseline, *R180* 3 h of reperfusion, *Δ *difference between R180 and *B*

* ANOVA for differences in the three groups in a single time point, ** *P* < 0.05 by post hoc tests versus control group

### Microvascular obstruction versus regional myocardial blood flow and no-reflow phenomenon

**Fig. 6:**
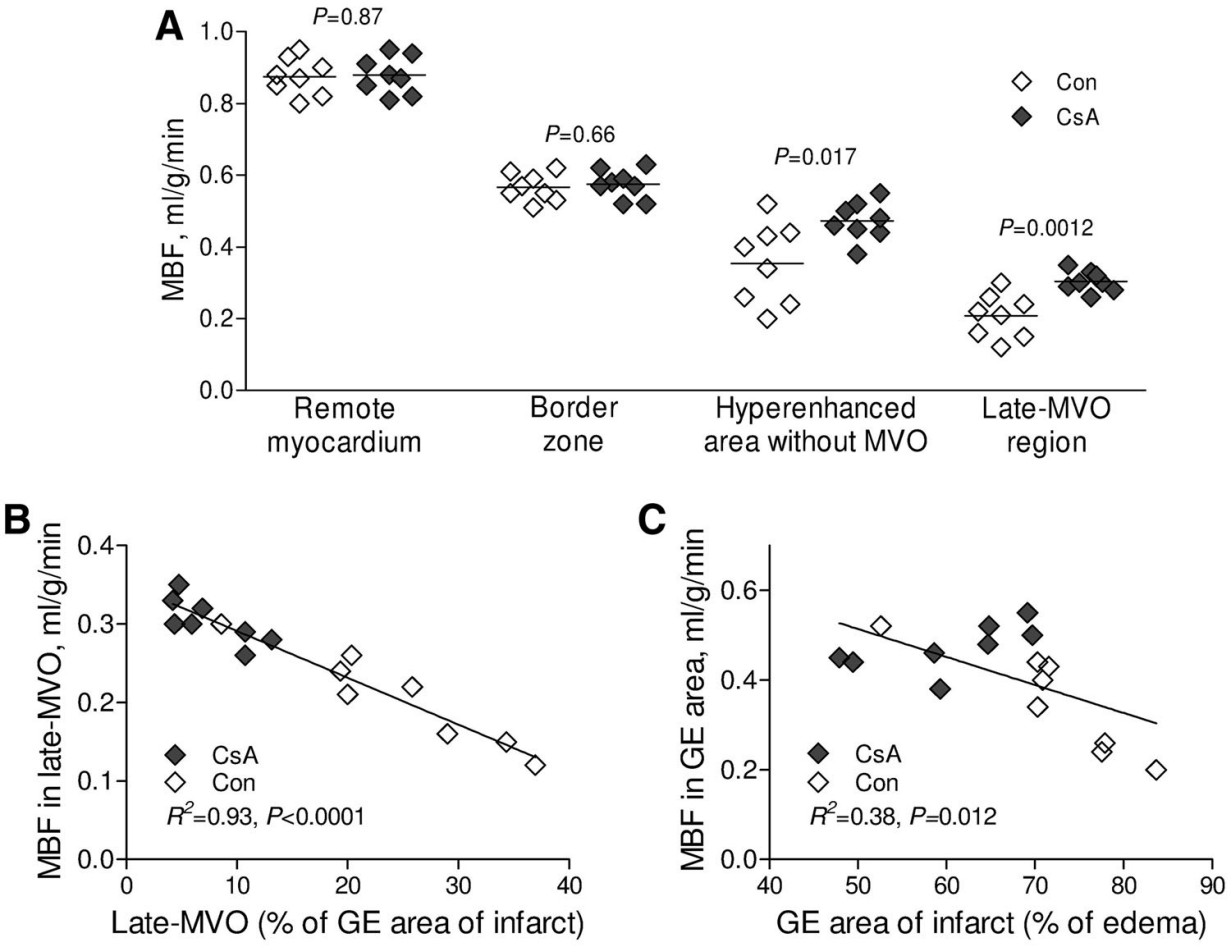
Myocardial blood flow. **a** Myocardial blood flow (MBF) in the territory of late microvascular obstruction (MVO), gadolinium-hyperenhanced area without MVO, border zone and in the remote myocardium. The relationship between **b** MBF in the region of late MVO and its size and **c** MBF in hyperenhanced area without MVO and the size of gadolinium-enhanced area of infarct. *Con* control group, *CsA* cyclosporine group

The myocardial edema was similar in both cyclosporine A and control pigs (48.3 ± 1.5 and 47.7 ± 1.9 %, respectively, *P* = 0.50) and did not correlate with the size of early MVO (*P* = 0.22), late MVO (*P* = 0.27) and the no-reflow area (*P* = 0.12). Cyclosporine A infusion significantly improved myocardial blood flow in the late MVO territory (0.30 ± 0.06 vs 0.21 ± 0.03 ml/g/min, *P* = 0.0012) and in the gadolinium-hyperenhanced infarct area (0.47 ± 0.05 vs 0.35 ± 0.11 ml/g/min, *P* = 0.017) as compared with control pigs. Cyclosporine A did not improve MBF in the border zone and in the remote myocardium (Fig. 6a). MBF in the region of late MVO was inversely correlated with the size of late MVO (Fig. 6b). There was also inverse correlation between MBF in the GE area of infarct and the size of GE area of infarct expressed as percent of myocardial edema (Fig. 6c).

**Fig. 7:**
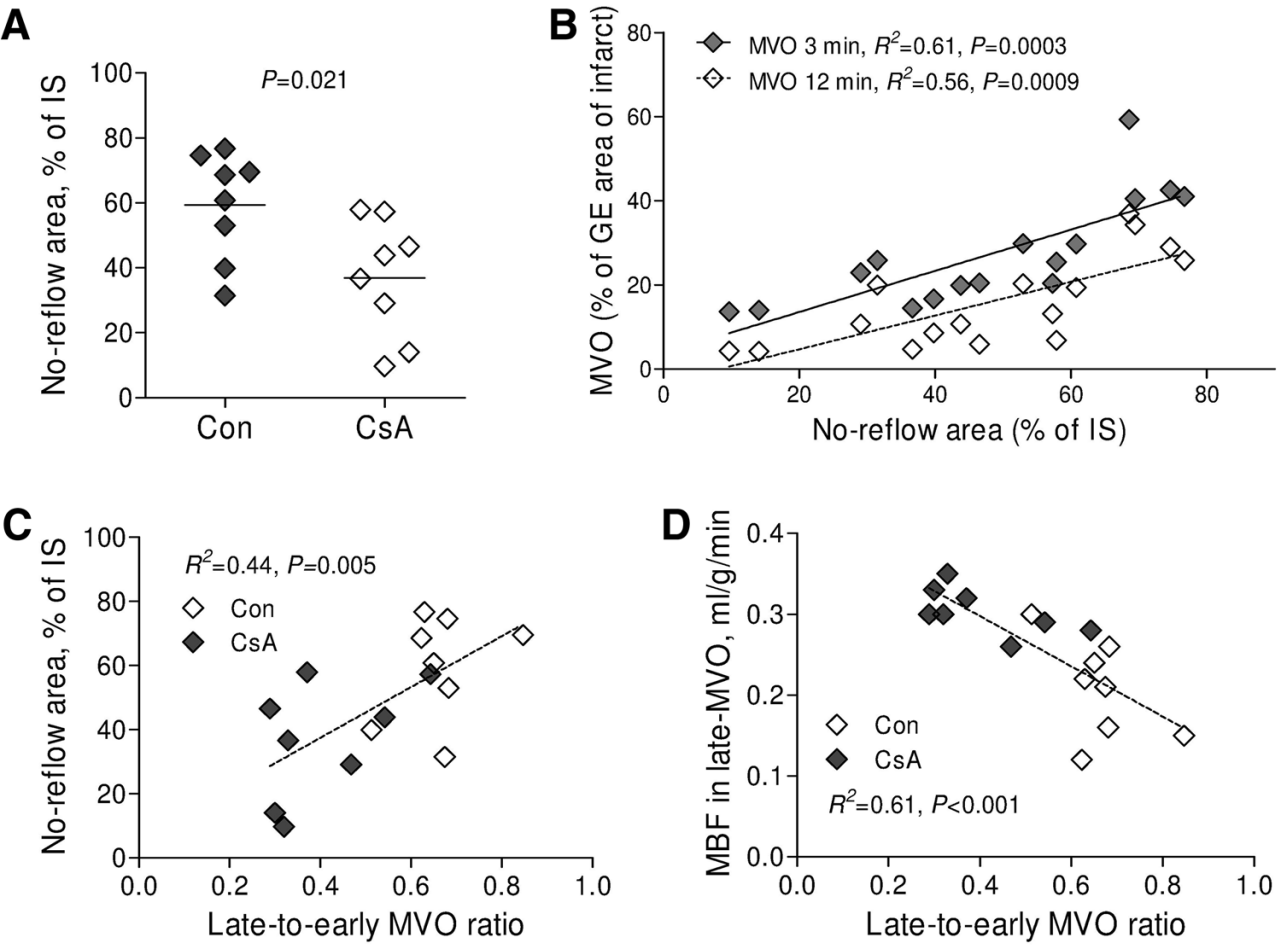
No-reflow area. **a** No-reflow area as measured by the lack of thioflavin S staining within infarct size (IS) as measured by triphenyltetrazolium staining. The relationship between no-reflow area and **b** microvascular obstruction (MVO) or **c** late-to-early MVO ratio and **d** the relationship between late-to-early MVO ratio and myocardial blood flow in the late MVO

The no-reflow area expressed as the area of lack of thioflavin S staining within the TTC territory was significantly lower in cyclosporine A group as compared with control pigs (36.9 ± 18.2 and 59.3 ± 16.6 %, respectively, *P* = 0.021, Fig. 7a) and was significantly correlated with the size of early- and late MVO (Fig. 7b). The ratio of late-to-early MVO shown in Fig. 4e was validated with the extent of no-reflow area and with the myocardial blood flow in the late MVO territory. This ratio was significantly correlated with no-reflow area (*R* ^*2*^ = 0.44, *P* = 0.005, Fig. 7c) and inversely correlated with MBF in the late MVO (*R* ^*2*^ = 0.61, *P* < 0.001, Fig. 7d).

### Histopathological findings

**Fig. 8:**
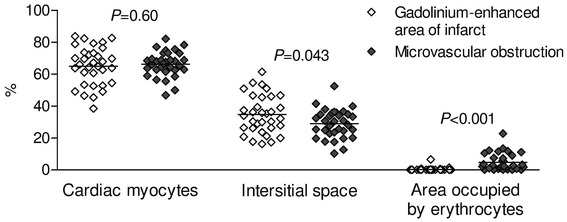
Histological composition of infarct area with and without microvascular obstruction. Data are shown as mean and absolute values

The microscopic analysis focused on loss of myocytes, interstitial edema and signs of capillary stasis. In the infarct zone, the three groups showed increased edema with reduced density of myocytes as compared with the remote myocardium. Similarly, capillaries filled with erythrocytes were observed in the infarct zone and only very rarely in the border zone and remote myocardium. These changes were seen in all pigs. No significant differences between the groups were found (Online Resource 2). In all pigs, microvascular obstruction area was associated with higher erythrocyte stasis (respective median (IQR) values are 2.4 (0.9–7.5) vs. 0 (0–0.05) %, *P* < 0.001) and lower interstitial edema [30.5 (23.5–34.8) vs. 34.0 (26.3–45.5) %, *P* = 0.043] as compared with hyperenhanced area (Fig. 8).

## Discussion

The current study demonstrates that cyclosporine A infusion just before restoration of epicardial blood flow protects against microvascular obstruction as measured in vivo by cardiovascular magnetic resonance imaging in a pig model of ischemia and reperfusion. Despite both postconditioning and cyclosporine A infusion significantly reduced infarct size and a total time of life-threatening ventricular arrhythmia, only cyclosporine A was associated with a better preserved left ventricular function following reperfusion. Moreover, cyclosporine A infusion significantly reduced no-reflow area and improved regional myocardial blood flow in the zone of microvascular obstruction and hyperenhanced area of infarct. Finally, both infarct size and microvascular obstruction area have been shown to be independent predictors of left ventricular function deterioration after coronary occlusion and reperfusion.

Postconditioning and cyclosporine A infusion are considered to have similar cardioprotective effects during I/R involving inhibition of mPTP opening and preservation of calcium retention capacity in cardiac myocytes [15–17]. Nevertheless, in contrast to postconditioning, cyclosporine A does not improve mitochondrial respiration [16]. Cyclosporine A, however, prevents opening of mPTP by inhibition of its interaction with cyclophilin D [3, 39]. Recently a meta-analysis of experimental studies shown that cyclosporine A reduces infarct size [35] but the results obtained in studies involving pigs were inconsistent possibly related to different doses of cyclosporine A (2.5–10 mg/kg) used [1, 29, 30, 34, 48]. Our data demonstrate that pretreatment with 10 mg/kg of cyclosporine A significantly limited infarct size but did not affect the extent of myocardial edema after 3 h of reperfusion. Our intention was to produce infarcts with as close as possible the same area at risk in all treated animals owing to occlusion of left anterior descending artery after the origin of the first diagonal branch. As a consequence area at risk as measured by T2-weighted edema in all examined pigs had small deviation of measurements from mean value. We have shown that edema was not limited by cyclosporine A as compared with controls. In contrast, Diaz et al. [10] in a model of I/R of rabbit cardiomyocytes in cell culture have found that cyclosporine A protects against cardiac myocyte necrosis by induction of chloride anions efflux from myocytes enhancing their cell-volume regulatory response without changes in transmembrane mitochondrial gradient. We found also that the extent of myocardial edema did not influence the size of microvascular obstruction and the no-reflow area. In turn, apart from maintaining acidosis, autacoids released during postconditioning activate survival signaling pathways including reperfusion injury salvage kinese pathway [21]. Previous postconditioning protocols in pigs were composed of 4–8 cycles of repeated 20–30-s of ischemia and reperfusion [25, 27, 47–49]. We did not use postconditioning protocol with shorter 20-s cycle of ischemia and reperfusion requiring open-chest dissection and cannulation of the coronary artery [25, 48, 49]. Our closed-chest catheter-based model of I/R does not allow rapid changes of postconditioning cycles because of the slowness of the balloon inflations and deflations. Another postconditioning protocol in pigs with 4 cycles of 30-s of ischemia and reperfusion did not limit infarct size [27] but 8 cycles of 30-s of ischemia and reperfusion elicited profound reduction of infarct size in Schwartz and Lagranha study [47] and was also effective in our closed-chest catheter-based model.

The mechanism of protection against coronary microvasculature damage associated with postconditioning or cyclosporine A and its influence on myocardial injury and left ventricular function remains poorly understood. Following I/R, microvascular perfusion defect despite patency of epicardial artery known as no-reflow phenomenon is first a consequence of extensive capillary damage with endothelial cell swelling, large membrane-bound intraluminal blebs of endothelial cells and cardiomyocyte swelling [32]. Plugging of leukocytes or erythrocytes, intraluminal fibrin thrombi, endothelial gaps with loss of fluid from capillaries and extravascular hemorrhage may also contribute to the microvascular obstruction [32, 46]. However, the effect of single, lasting a few minutes infusion of cyclosporine A or several postconditioning cycles on endothelial cell function and structure, activation of circulating inflammatory cells and on properties of intraluminal thrombus during acute phase of myocardial infarction has not been examined so far. Massoudy et al. [37] have shown that in a model of I/R of isolated guinea-pig heart, cyclosporine A improved myocardial post-ischemic function by enhancing nitric oxide release from endothelium with simultaneous reduction of oxidative stress. In turn, protection with postconditioning against I/R injury of human endothelium in the brachial artery and resistance vessels depended on K_ATP_ channel activation and was mimicked by cyclosporine A [41]. In our model of I/R of porcine myocardium cyclosporine A infusion but not postconditioning reduced significantly microvascular obstruction. Simultaneously, cyclosporine A improved myocardial blood flow in the late MVO and reduced the extent of no-reflow phenomenon. We have shown also that the larger the late microvascular obstruction, the lower myocardial blood flow in the late MVO as measured by CMR and the bigger the no-reflow territory determined by the lack of thioflavin S staining.

Both cyclosporine A infusion and postconditioning accelerated the reduction of microvascular obstruction between 3 and 12 min after contrast injection, suggesting a faster contrast penetration due to more preserved microvascular patency and finally better reperfusion. However, the effect was more pronounced in cyclosporine A-treated pigs. The ratio of late-to-early MVO validated with the extent of no-reflow area and with the myocardial blood flow in the late-MVO territory, reflects the dynamics of contrast penetration throughout coronary microcirculation during the early phase of reperfusion. We found that the lower the ratio of late-to-early MVO the smaller the no-reflow area and the better myocardial blood flow in late-MVO territory.

The cause–effect relationship between myocardial and microvascular injury is still a matter of debate. Experimental studies indicate that structural changes in the no-reflow microvasculature are preceded by and limited to the area of myocyte necrosis [32, 33]. Those findings suggest that the microvascular damage is not a primary reason of changes in cardiac myocytes. Simultaneously, no-reflow regions preceded the presence of macroscopically visible hemorrhage in the infarct region [13, 32, 45], however, the influence of damaged microvasculature on myocardial necrosis remains unclear. On the other hand, myocardial perfusion in the area at risk, both after 30 as well as 120 min of ischemia, progressively decreased within the first 2 h of reperfusion reaching a plateau at about 50 and 30 % of normal perfusion, respectively [44]. Simultaneously, the extent of no-reflow as measured by the lack of thioflavin S dye within area at risk increased over the course of reperfusion from 12 % after 2 min to 31 % after 2 h and 35 % after 8 h [45]. Moreover the no-reflow extent was correlated with infarct size after 1 h of reperfusion [45] and inversely correlated with residual myocardial perfusion after 4 h [28]. We also observed—irrespective of treatment assignment—a significant positive correlation between infarct size and microvascular obstruction or no-reflow area, nevertheless our results do not provide arguments what comes first.

In the clinical settings, infarct size and microvascular obstruction are important prognostic factors for cardiovascular outcome [42, 53]. Their simultaneous predictive value in determination of left ventricular function recovery after myocardial infarction is inconsistent. The LVEF at 6-month follow-up in STEMI patients was independently predicted by myocardial infarct size but not MVO area [4]. In contrast, Wong et al. [52] have shown that only late MVO was the independent predictor of LVEF at 90 days. It was also found that both infarct size and microvascular obstruction predict LVEF in 3–4-month follow-up [12, 40]. Our findings indicate that both infarct size as well as microvascular obstruction independently and significantly affected deterioration of LVEF immediately after coronary occlusion and reperfusion. Moreover, the importance of infarct size for the prediction of functional deterioration in acute phase is almost four times bigger than associated with microvascular obstruction, but the additive protective effect on both components visible with cyclosporine A infusion but not with postconditioning was mandatory for the effective preservation of left ventricular function.

Our study also allowed comparing cardiovascular magnetic resonance measurements with pathology findings. We have found that infarct size measured in vivo by gadolinium enhancement strongly correlated with triphenyltetrazolium-determined area of infarct. Recently, Robberts et al. [46] have shown that the contrast-devoid core of infarct 7 days after induction of myocardial infarction in pigs contained extensive necrosis with erythrocytes extravasation. We have shown that immediately after 3 h of reperfusion in the triphenyltetrazolium-positive infarct region erythrocyte stasis was significantly higher in biopsies corresponding with a low-signal intensity region of microvascular obstruction as compared with hyperenhanced area. These histopathological differences are primarily the consequence of specific dynamic changes in the infarct area involving the evolution over time of myocardial edema and extent of microvascular obstruction [42].

It was shown that postconditioning with 4 cycles of 1-min ischemia and reperfusion prior to primary coronary angioplasty in patients reduces infarct size [51] and attenuates microvascular obstruction [38]. However, in both studies postconditioning patients had less myocardial edema suggesting a lower area at risk at baseline. Moreover, in 60 % of the patients postconditioning was preceded by aspiration thrombectomy rapidly restoring epicardial flow and abolishing pH-related protective effects of postconditioning. In contrast, the same postconditioning protocol tested in two randomized trials with cardiovascular magnetic resonance endpoints [14, 50] was not able to reduce gadolinium-enhanced infarcted area measured 6–9 days and 6 months after primary angioplasty. Recently, in the largest randomized clinical trial to date [18] postconditioning did not improve ST-segment resolution, myocardial blush and 30-day clinical outcome. In turn, four or more inflations during PPCI reduced enzymatic infarct size in STEMI patients, but did not translate into improved 4-year long-term outcomes as compared with patients receiving 1–3 inflations in the infarct-related artery [55]. Small randomized clinical studies showed that cyclosporine infusion before the opening of infarct-related artery [43] or after induction of anesthesia for elective cardiac artery bypass surgery [20] resulted in a significant reduction of enzymatic infarct size compared with controls. Our results suggest that beyond infarct size reduction, the beneficial effect of cyclosporine A may also be related to better regional myocardial blood flow and preserved left ventricular function.

In conclusion, in our animal model of ischemia and reperfusion both cyclosporine A and postconditioning reduce infarct size, however, only cyclosporine A infusion had a beneficial effect on microvascular damage and was associated with better preserved LV function when compared with controls. Our findings indicate that only simultaneous limitation of myocardial and microvascular injury is associated with functional improvement, therefore, we suggest the use of microvascular obstruction as a surrogate endpoint in future studies in addition to infarct size.

## Electronic supplementary material

Below is the link to the electronic supplementary material.

Supplementary material 1 (PDF 31 kb)

Supplementary material 2 (PDF 97 kb)
