# Supplementary material for: Cyclosporine A reduces microvascular obstruction and preserves left ventricular function deterioration following myocardial ischemia and reperfusion
Source: Basic Res Cardiol. 2015 Feb 27;110(2):18. doi: 10.1007/s00395-015-0475-8 (PMC4342514; doi:10.1007/s00395-015-0475-8)
Supplement: Supplementary file 1 — Supplementary material 1 (PDF 31 kb) [file 395_2015_475_MOESM1_ESM.pdf]

Online Resource 1. The comparison of the area at risk and left ventricular ejection fraction in both stages of the study.

	Baseline experiments				Additional experiments		
Study group	CsA	PoC	Con	*P*-value of ANOVA for CsA vs. PoC vs. Con	CsA	Con	*P*-value CsA vs. Con
Area at risk, %*	50.2±1.6	49.8±1.9	49.4±2.6	0.77	48.3±1.5	47.7±1.9	0.50
Left ventricular ejection fraction at 3h of reperfusion, % ***	46.3±3.5**	42.9±5.9	38.9±5.6	0.036	44.1±2.9	36.3±3.7	<0.001

Abbreviations: Con: control group, PoC: postconditioning group, CsA: cyclosporine A group.

* At baseline determined by Evans blue staining as percentage of left ventricle, in additional experiments by T2-weighted myocardial edema in cardiac magnetic resonance imaging as percentage of left ventricle.

** P=0.005 by post-hoc test versus control group.

*** P=0.35 for differences between baseline and additional experiments in terms of treatment effect associated with cyclosporine A infusion.
