# Supplementary material for: Cyclosporine A reduces microvascular obstruction and preserves left ventricular function deterioration following myocardial ischemia and reperfusion
Source: Basic Res Cardiol. 2015 Feb 27;110(2):18. doi: 10.1007/s00395-015-0475-8 (PMC4342514; doi:10.1007/s00395-015-0475-8)
Supplement: Supplementary file 2 — Supplementary material 2 (PDF 97 kb) [file 395_2015_475_MOESM2_ESM.pdf]

Online resource 2. Microscopic characteristics of compared groups.

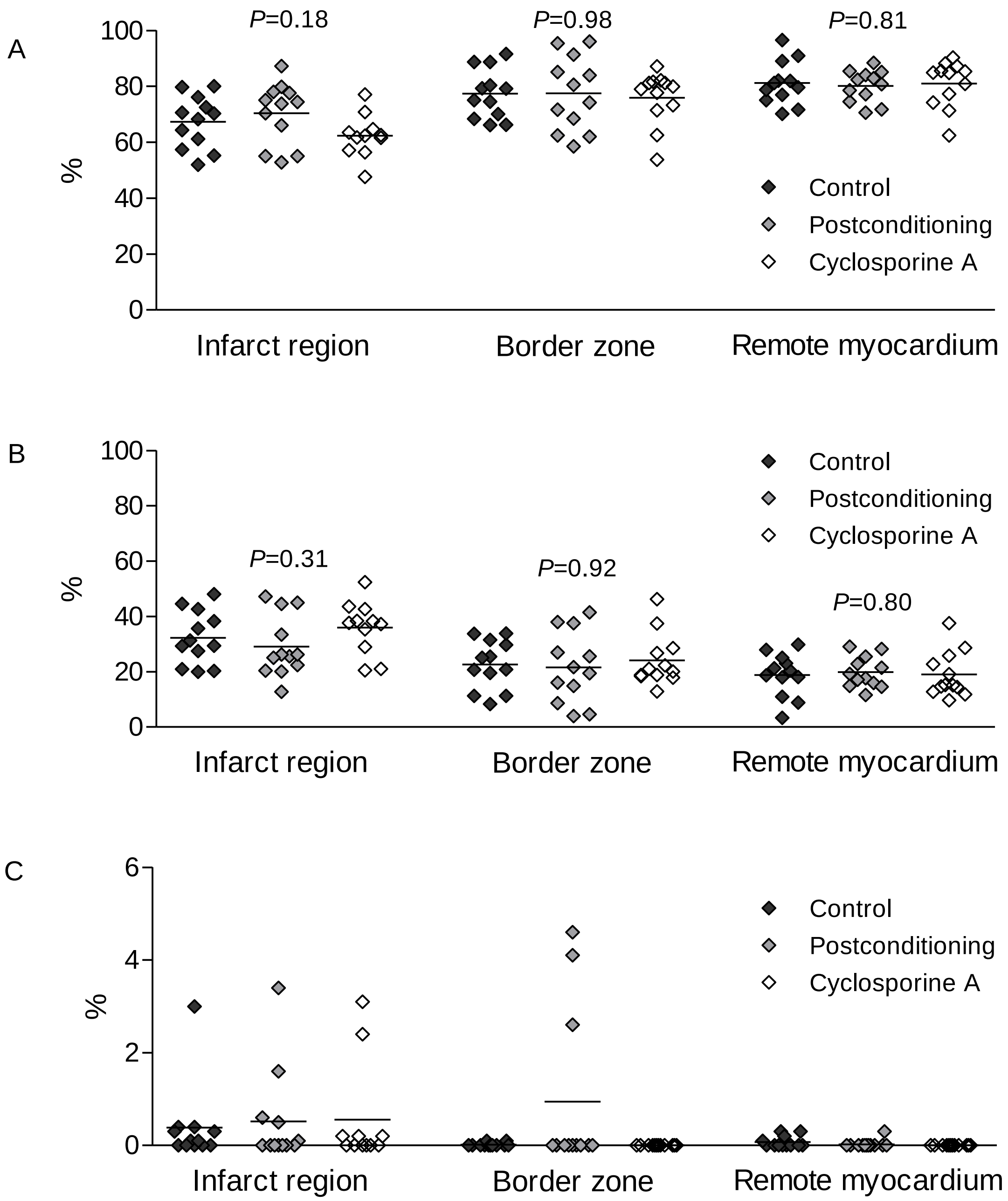

Abbreviations: data are shown as mean and absolute values. A. Cardiac myocytes, B. Interstitial space, C. Area occupied by erythrocytes.
